# Venous Thromboembolism in Inherited Platelet Disorders: A Clinical Challenge

**DOI:** 10.3390/diagnostics15212667

**Published:** 2025-10-22

**Authors:** Francesco Paciullo, Patrizia Rovere-Querini, Loredana Bury, Emanuela Falcinelli, Paolo Gresele

**Affiliations:** 1 Faculty of Medicine and Surgery, Vita-Salute San Raffaele University, 20132 Milan, Italy; rovere.patrizia@hsr.it; 2 Unit of Internal Medicine, Division of Immunology, Transplantation, and Infectious Diseases, IRCCS San Raffaele Scientific Institute, 20132 Milan, Italy; 3 Department of Medicine and Surgery, Section of Internal and Cardiovascular Medicine, University of Perugia, 06125 Perugia, Italy; loredana.bury@unipg.it (L.B.); emanuelafalcinelli@gmail.com (E.F.); paolo.gresele@unipg.it (P.G.)

**Keywords:** inherited platelet disorders, venous thromboembolism, thromboprophylaxis, bleeding risk

## Abstract

Inherited platelet disorders (IPDs) are rare hematological conditions characterized by abnormal platelet function or number, predisposing patients to bleeding. Even if they apparently lower the risk of venous thromboembolism (VTE), this is not abolished in these patients, and may represent a potential cause of mortality. VTE prevention and treatment in these patients is particularly challenging due to the delicate balance between thrombosis and bleeding risks. Here, we summarize current evidence on the incidence, risk factors, and management strategies for VTE in IPD patients, with a focus on the perioperative setting.

## 1. Introduction

Inherited platelet disorders (IPD) include rare congenital bleeding diseases characterized by abnormal platelet function (IPFD) and/or number (IPND), leading to an increased bleeding risk [1,2]. While IPND diagnosis relies mainly on history, blood count, and blood smear evaluation, IPFD diagnosis is more complex and requires specialized platelet function tests, often unavailable in non-specialized laboratories. According to the International Society of Hemostasis and Thrombosis (ISTH) guidelines, IPD should be considered in patients with unexplained bleeding and normal basal hemostatic tests, especially when a familIial history of bleeding diathesis is present [3].

Venous thromboembolism (VTE) is a potentially life-threatening condition that requires anticoagulant therapy, which increases bleeding risk. Although platelets play a role in coagulation, IPD patients may still develop VTE, particularly in high-risk situations, such as surgery or prolonged immobilization. The management of VTE in these patients is challenging due to the prohemorrhagic effects of therapy, and currently, no specific guidelines exist.

This review aims to discuss the critical aspects of VTE in patients with platelet disorders and suggest the best approaches to management in this complex setting.

## 2. Background on Thrombotic and Bleeding Risks in VTE Management

VTE is a common and potentially life-threatening condition, with an estimated incidence of 1–2 cases per 1000 inhabitants and an in-hospital mortality of 3–4% [4]. Most VTE events originate in the deep veins of the lower limbs but can also involve other sites, such as cerebral, splanchnic, or upper extremity veins. The clinical impact and mortality of VTE are influenced by factors such as the extent of embolization, presence of cancer or comorbidities, and age; for example, mortality is <2% in isolated deep vein thrombosis (DVT) but increases with age, neoplasms, or massive pulmonary embolism [5]. Major bleeding events during anticoagulation further worsen prognosis [6]. The risk of VTE recurrence depends on the provoking factor and ranges from 10% at one year and 30% at five years in unprovoked cases, to 20% at one year in cancer patients, and <3% at five years in VTE associated with transient risk factors [7]. Moderate-high risk thrombophilia increases recurrence risk [8]. Management is based on balancing recurrence and bleeding risks, a dilemma especially relevant in patients with inherited bleeding disorders. Surgery is a major transient risk factor for VTE, with incidence rates up to 20% in general surgery and 60% in orthopedic surgery without prophylaxis [9,10]. The ACCP guidelines stratify surgical procedures into three VTE-risk categories, and the Caprini score is widely used for individual risk assessment [11,12]. This is crucial in IPD patients, where both thrombotic and bleeding risks must be carefully weighed [13,14]. Anticoagulation for VTE is associated with an enhanced risk of major bleeding, with incidences of 3.86, 2.93, and 1.95 per 100 patient-years for warfarin, rivaroxaban, and apixaban, respectively, decreasing with reduced doses of direct oral anticoagulants (DOACs) during extended treatment [15]. Comparative studies show that DOACs and aspirin have similar risks of intracranial hemorrhage, but major bleeding is more frequent with DOACs, especially rivaroxaban [16]. For surgical patients on anticoagulation, the perioperative bleeding risk should be assessed clinically based on the procedure type and patient factors, as recommended by the 2022 ACCP guidelines, which categorize procedures into low, intermediate, or high bleeding risk [17]. This individualized approach is essential in complex patients such as those with IPD.

## 3. Role of Platelets in VTE

**Figure 1:**
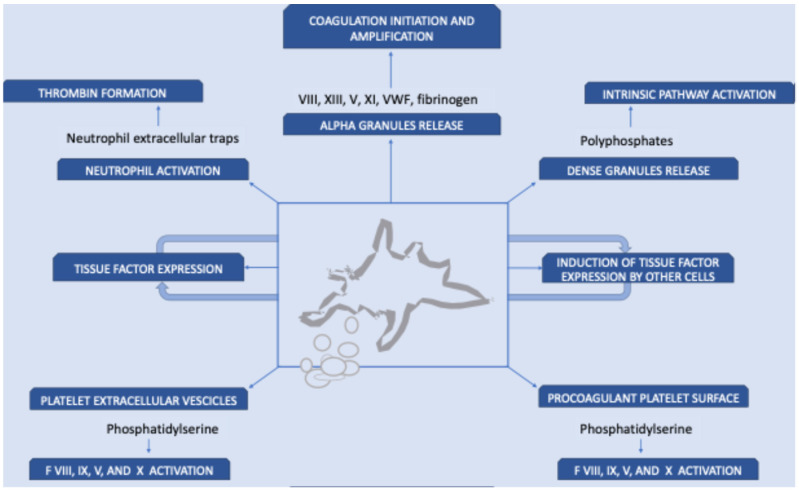
Platelet role in coagulation. The figure summarizes the mechanisms through which platelets influence coagulation. Platelets release several coagulation factors—including factor VIII, V, XI, XIII, von Willebrand factor, and fibrinogen—from their alpha granules. In addition, they express tissue factor on their surface and stimulate its expression by other inflammatory cells. Platelets also shed extracellular vesicles containing procoagulant substances and expose phosphatidylserine, thereby providing a catalytic surface for the initiation of the coagulation cascade. Furthermore, platelets interact with neutrophils, inducing the release of neutrophil extracellular traps (NETs), which, in turn, activate blood clotting through various mechanisms and activate the intrinsic pathway of coagulation through the release of polyphosphates from their dense granules.

Although venous VTE is classically considered a condition driven mainly by hypercoagulability—unlike arterial thrombosis, where platelets are the central effectors—recent evidence highlights that platelets also play a significant and multifaceted role in VTE pathogenesis (Figure 1).

In particular, platelets directly activate blood coagulation by releasing clotting factors, such as factor V, and expose upon activation phosphatidylserine on their membrane, which represents one of the most relevant sites of binding and activation of the tenase and prothrombinase complexes [18]. Through these mechanisms, platelets provide a catalytic surface that amplifies the coagulation cascade. In addition, activated platelets release polyphosphates from their dense granules, initiating the intrinsic coagulation pathway [19,20] and inducing the expression of tissue factor by other cells [21]. This cross-talk further enhances the prothrombotic environment. Moreover, extracellular vesicles (EVs) derived from activated platelets exhibit a phospholipid-rich biomolecular profile, with particularly high levels of phosphatidylserine, which confer strong procoagulant properties. These EVs promote the assembly and activity of coagulation complexes, such as the prothrombinase complex, leading to a significant increase in thrombin generation [22], also through the expression of tissue factor. Platelets are also able to induce tissue factor expression by other cells, including macrophages [23]. Another key aspect is the interaction of platelets with the innate immune system: activated platelets induce neutrophil activation and the subsequent release of DNA histone complexes, called neutrophil extracellular traps (NETs), which, in turn, can activate coagulation and thrombin formation [24] (Figure 1). Altogether, these findings illustrate how platelets may contribute to VTE not only as passive bystanders but as active players in the thrombotic process.

## 4. VTE in IPD Patients

Given these platelet-mediated mechanisms, one might expect that a platelet dysfunction would impair blood clotting activation and thus offer some protection from VTE. Accordingly, in a mouse model of FeCl3-triggered inferior vena cava thrombosis, the absence of GPIb-V-IX (the defect associated with Bernard-Soulier syndrome) prevented the development of thrombosis [25]. However, the situation appears more complex in a clinical setting. For instance, platelets from Glanzman thrombasthenia (GT) patients have been found to expose fibrin binding sites and to be able to support normal thrombin generation [26]. Moreover, although the real incidence of VTE in the IPD patient population remains unknown, cases of thrombosis have been reported in several subjects with IPD (Table 1), supporting the idea that platelet dysfunction does not necessarily affect the platelet procoagulant proprieties [27,28]. This suggests that, despite their bleeding tendency, these patients may not be fully protected from thrombotic events. Moreover, it has to be noted that IPD patients may be exposed to the same VTE risk factors as the general population, including neoplasms, immune diseases, surgery, immobilization, thrombophilia, and prothrombotic drugs [29]. In particular, being more prone to be treated with prohemostatic agents, especially in the case of active bleeding or in the perioperative setting, IPD patients may be exposed to the thrombotic risks associated with these drugs [30].

## 5. Pro-Hemostatic Agents Used in IPD and Their Thrombotic Risk

### 5.1. Platelet Concentrates

Platelet concentrates (PC) are often administered to prevent or treat spontaneous or traumatic bleeding and as prophylaxis before, during and after surgery [31,32]. In a study, including more than 500,000 neoplastic hospitalized patients, the use of PC was associated with a significantly increased risk of venous and arterial thrombosis (OR: 1.20; 1.11–1.29). Such risk is supposed to be reduced using leuko-reduced concentrates containing low levels of platelet microvesicles, the most important procoagulant mediators of platelets [33]. In the surgical setting, among IPD patients, the use of PC is reported to occur in almost 40% of cases, more frequently for the prophylaxis of major surgery and after post-surgical bleeding [32], but no data are available on its potential prothrombotic risk.

### 5.2. Recombinant Activated FVII

Recombinant activated FVII (rFVIIa) is currently approved for the management of bleeding episodes in patients with FVII deficiency, hemophilia with inhibitors and in GT, but it is sometimes used off-label in other forms of IPD [34]. The rationale for the use of rFVIIa in IPD patients is related to the enhanced rate of thrombin generation that it induces on the surface of thrombin-activated platelets [35]. In patients with GT, the overall incidence of thrombosis reported in clinical trials and registers has been of 0.19%, increasing with older age, concurrent cardiac and vascular disease and use of prothrombin complex concentrates [36].

### 5.3. Desmopressin

Desmopressin is one of the most widely prescribed antihemorrhagic agents among IPD patients [37,38]. Its mechanism of action is related to its ability to release von Willebrand factor from endothelial cells, potentiating platelet adhesion and aggregation. A systematic review and meta-analysis of four trials reporting the outcome of thrombotic events in patients treated with desmopressin for platelet dysfunction found no increase in the risk of VTE (OR 0.56 95% CI, 0.06–5.50) [39].

### 5.4. Antifibrinolytic Agents

Antifibrinolytic drugs, including tranexamic acid (TXA) and aminocaproic acid, are a class of antihemorrhagic agents that act by inhibiting endogenous fibrinolysis. Clinical trials have reported an overall 25% reduction in bleeding events when used in the perioperative period during high bleeding risk surgery [40], with a non-significant effect in terms of thrombotic risk.

However, a recent review and meta-analysis of 57 studies evaluating the effects of antifibrinolytic drugs in patients with spontaneous bleeding reported a significant increase of VTE with the use of TXA (1.9%; 95% CI; 1.1 to 2.9) and with epsilon aminocaproic acid (EACA) (3.0%; 95% CI 1.8 to 4.6) [41]. A subsequent meta-analysis though, including 22 studies with 49,538 medical patients treated with TXA, reported no increased risk of pulmonary embolism (RR = 0.97; 95% CI = 0.75–1.26; I2 = 0%), or deep vein thrombosis (RR = 0.99; 95% CI = 0.70–1.41; I2 = 0%) compared to control [42].

### 5.5. Thrombopoietin Receptor Agonists

Thrombopoietin (TPO) is a glycoprotein secreted by the liver that acts on a receptor on megakaryocytes, favoring their proliferation and differentiation into platelets. TPO receptor agonists (TPO-RA) are a class of molecules that mimic TPO activity, favoring the rise in platelet count. Romiplostim is a peptibody that binds directly and competitively to the TPO binding site of the TPO-R, whereas eltrombopag and avatrombopag are small molecules that bind at a transmembrane site. Currently, the use of TPO agonists is approved for immune thrombocytopenia; nevertheless, several recent reports suggest their possible use in some IPD, such as the Wiskott–Aldrich syndrome, MYH9-related disorder, ANKRD26-related thrombocytopenia, and the GPIIb/IIIa- related thrombocytopenia [43]. TPO-RA can be administered in preparation for elective surgery and/or invasive procedures or prior to hematopoietic stem cell transplantation in cases of severe thrombocytopenia, such as WAS [44]. The most feared complication of TPO therapy is represented by thrombosis, particularly if prothrombotic conditions concur, such as the antiphospholipid syndrome [45,46,47,48]. Anyway, in 4 published studies of the use of TPO RA in IPD, only one case of arterial thrombosis with romiplostim was reported out of 112 treated patients [49,50,51].

### 5.6. Red Blood Cell Transfusions

Red blood cells (RBCs) are often required in surgical patients, especially in those with IPD, and are associated with an increased risk of VTE ranging from 1 to 5% [52].

## 6. VTE in IPD Patients: Prevention and Management

The incidence of VTE in IPD patients undergoing surgical procedures at risk of thrombosis is unknown, and no clinical trials have been reported so far. Moreover, surgery exposes patients to bleeding, which makes the use of antithrombotic thromboprophylaxis in subjects presenting with a preexisting hemostatic disease a challenge. No systematic studies on the use of thromboprophylaxis in IPD patients undergoing surgery have been carried out so far, and little information on the safety of the prophylactic administration of LMWH to IPD patients is available, although isolated reports on the administration of anticoagulants to IPD patients have been published, suggesting relative safety [30,31]. In the population of the SPATA registry, a large retrospective study on the complications of surgery in IPD patients, only 2 VTE events were reported among 155 patients with IPD undergoing surgery, both events occurring in patients at high VTE risk and not undergoing thromboprophylaxis [30]. Similar to the general population, IPD surgery may also significantly enhance VTE risk, especially considering that patients are often exposed to prohemostatic agents for the preparation of the invasive procedure or for postoperative bleeding management, including antifibrinolytics, TPO mimetics, rFVIIa, platelet concentrate transfusions, and desmopressin [32]. In a recent review, A.T. Nurden reported 9 VTE cases in patients with GT (in 3 of whom recurrent), all occurring in the presence of concomitant VTE risk conditions, including congenital thrombophilia, immobilization, and the use of prohemostatic agents. Surgery was recognized as the main prothrombotic risk factor in three of these cases [29]. Overall, these data emphasize the need for individualized assessment and multidisciplinary management in IPD patients undergoing surgery.

## 7. Use of Anticoagulants in IPD: Balancing Risks and Benefits

Managing anticoagulation in IPD is a clinical challenge that goes beyond simply weighing thrombosis against bleeding, often due to the lack of standardized guidelines and taking into account highly individualized scenarios. In the general population, the lifelong risk of major bleeding increases with age and is higher in men, ranging from 1.8 to 6.4 per 1000 person-years [53]. Selak et al. recently developed predictive models for major bleeding, showing that risk is influenced by several patient-specific factors [54]. Main predictors included older age, male sex, low hemoglobin, prior bleeding, cancer, peptic ulcer, liver disease or pancreatitis, alcohol-related conditions, and use of medications increasing bleeding risk (such as NSAIDs, antiplatelet agents). In IPD, these models alone are often insufficient, as the bleeding phenotype depends also on the underlying genetic defect (Table 2), platelet count, and the personal bleeding history [55,56]. Nonetheless, even knowing the exact platelet disorder does not allow precise prediction of the hemorrhagic risk [57,58]; moreover, thrombocytopenia alone does not linearly predict bleeding risk. A potential additional tool to optimize individual bleeding risk assessment in these patients is the ISTH BAT, a multiparametric score that evaluates the likelihood of an inherited bleeding disorder based on the patient’s bleeding history, originally developed for von Willebrand disease [55] and recently validated in IPD patients [56]. The cumulative score helps clinicians to distinguish between clinically significant and minor bleeding, supporting diagnosis and management decisions in both inherited and acquired bleeding disorders, while it does not seem to be useful as a screening test in the general population to predict future bleedings or among patients with bleeding diseases of unknown cause [59].

Recently, several tools have been developed to estimate bleeding risk in patients on anticoagulation in different settings [60]. Among VTE patients, the VTE-BLEED score has shown good predictive power for major bleeding and can identify those at high risk of intracranial and fatal bleeding [60]. Beyond platelet count, the history of bleeding, age, use of anticoagulants/antiplatelets, comorbidities, infections, recent surgery, and risk behaviors are considered for bleeding risk; personal thrombotic history, cardiovascular risk factors, immobility, thrombopoietin receptor agonists, antiphospholipid antibodies, hormonal therapies, and malignancies for thrombotic risk. Patients are classified into high, intermediate, or low risk for both bleeding and thrombosis, with regular reassessment recommended to tailor therapy [61]. Even if not validated in IPD, the use of the VTE-BLEED score may contribute to improving the sensitivity of individual bleeding risk prediction even in this population when anticoagulation is indicated [60]. Moreover, even the WHO bleeding score, which categorizes bleeding severity, may also be useful in IPD [60]. Indeed, in the SPATA study, the preoperative WHO bleeding score was the best predictor of excessive perioperative bleeding, independent of thromboprophylaxis [30].

### 7.1. Thromboprophylaxis in IPD Patients

Since VTE prevention may significantly reduce the risk of VTE and thus reduce the need for full-dose anticoagulation, its use may be crucial in IPD patients.

VTE risk can be effectively mitigated by either mechanical or pharmacologic thromboprophylaxis. Mechanical thromboprophylaxis includes the use of compressive stockings or intermittent pneumatic compression devices. While its benefit in the prophylaxis of medical patients remains debated [62], in surgical patients, mechanical thromboprophylaxis has been reported to reduce VTE risk by 64% (intermittent pneumatic compressive device) and 60% (compressive stockings) [63,64]. Mechanical thromboprophylaxis has no effect on hemostasis and thus on bleeding incidence. Pharmacologic thromboprophylaxis does not influence the risk of major bleedings in the medical setting [65], while it just marginally increases it in surgical patients [66]. On the other hand, pharmacologic thromboprophylaxis, mainly performed with low-dose low-molecular-weight heparin (LMWH), reduces VTE risk by 75% compared with placebo in the general population. The addition of mechanical thromboprophylaxis does not seem to provide further benefit in terms of VTE prevention [67].

No systematic data are available on the use of thromboprophylaxis in IPD patients. However, a recent sub-study from the large SPATA registry reported a low prevalence of thromboprophylaxis use in IPD patients undergoing surgery (23.3% of procedures), with higher prevalence in orthopedic and gynecological surgeries.

All patients were retrospectively classified for their VTE risk according to the Caprini score. Among 210 procedures, thromboprophylaxis was adopted in only 49 patients, and in only 19 cases was it pharmacological, while in all the others it was mechanical. Of the 49 interventions managed with thromboprophylaxis, 13 were orthopedic (26.0%), 12 gynecological (24.5%), 7 abdominal (14.3%), 7 thoracic (14.3%), 7 urological (14.3%), and 3 neuro-spinal (6%). LMWH prophylaxis was adopted in 22% of the orthopedic procedures, 12.7% of gynecological, 11% of thoracic, 11% of neuro-spinal surgery, 7.1% of urological, and 4.2% of abdominal procedures. For the procedures at high VTE-risk according to the Caprini risk stratification, thromboprophylaxis was adopted in 33.8% (LWMH in 14, mechanical in 6, and both in two) with no VTE events, while in 43 it was not adopted. Regarding procedures at intermediate VTE-risk (*n* = 60), thromboprophylaxis was used in 15 (25%) (mechanical in 11 and pharmacologic in 4), while of the procedures at low VTE-risk (*n* = 53) thromboprophylaxis was used in 10 (18.9%) (9 mechanical, 1 both mechanical and pharmacologic), and for the procedures at very low VTE-risk (*n* = 32), thromboprophylaxis was used in only 2 patients (6.2%) (1 mechanical, 1 pharmacologic). According to the procedure-related VTE-risk stratification, 35 high-risk procedures, 114 intermediate-risk and 61 low-risk procedures were performed. Thromboprophylaxis was adopted in 42% (nine pharmacologic and six mechanical) of the high-risk procedures, in 21% (six pharmacologic, 15 mechanical and three both) of the intermediate-risk and in 16.4% (four pharmacologic and six mechanical) of the low-risk procedures. The choice of using LMWH was significantly associated with the Caprini risk class (*p* < 0.001) and with the procedure-related VTE-risk class (*p* < 0.05). VTE was not observed in patients undergoing mechanical or pharmacologic thromboprophylaxis. In the whole population, two VTEs were observed.

The two thrombotic events occurred in patients at the highest class of VTE risk, according to their Caprini score, and neither had undergone thromboprophylaxis, reflecting the relevance of VTE prevention also in IPD patients. Due to its favorable profile in terms of net clinical benefit, therapeutic thromboprophylaxis is largely accepted even in cases of underlying bleeding diseases unless the bleeding risk is extremely high (i.e., thrombocytopenia with platelet count < 25 × 10^9^/L) [68,69,70].

The use of pharmacologic thromboprophylaxis was not associated with excessive surgical or post-surgical bleeding, even in patients with a high basal bleeding risk [33]. These data suggest that the administration of thromboprophylaxis among IPD patients is relatively safe.

### 7.2. Anticoagulation in IPD Patients

Full anticoagulation represents the mainstay of treatment in patients with VTE as it is associated with a significant reduction in mortality and risk of recurrence. However, anticoagulated patients with PE still experience a 10% mortality at 14 days, in 6% of cases associated with severe hemorrhagic events [71]. The use of anticoagulation in patients at high bleeding risk represents a challenge as it adds the risk of anticoagulation to the intrinsic bleeding risk associated with the basal patient condition. Currently, no data are available to support clinicians in managing antithrombotic therapy in IPD patients; however, if carefully conducted the use of anticoagulation seems feasible [30]. In this setting, DOACs and, particularly, apixaban may represent the agent of choice due to their better safety profile [72,73]. Moreover, a low-dose regimen, normally reserved for the extended anticoagulation phase, may be considered for these patients in the acute phase, especially in those subjects at higher basal bleeding risk.

## 8. Use of Antithrombotic Therapy in Patients with Other Inherited or Acquired Bleeding Disorders

In recent years, there has been a growing body of expert consensus documents addressing the management of antithrombotic therapy in patients with bleeding disorders. This is particularly relevant for the treatment of VTE in patients with hemophilia, where the balance between bleeding and thrombotic risk is especially challenging. Current expert recommendations suggest that in hemophilic patients with VTE anticoagulation should be administered together with concomitant hemostatic support, aiming to maintain factor levels above a minimum threshold (for example factor VIII or IX levels >20–30% during the initial treatment phase and >5–10% during maintenance phase), with an individualized management approach based on the severity of hemophilia and bleeding/thrombotic risk [74]. Similarly, the management of VTE in patients with severe immune thrombocytopenia (ITP) presents significant challenges due to the heightened risk of bleeding associated with low platelet counts. The recent literature, including case series and reviews, suggests that anticoagulation can be considered in ITP patients with VTE when the platelet count is above 30–50 × 10^9^/L, ideally in combination with therapies aimed at increasing platelet levels, such as corticosteroids, intravenous immunoglobulins, or thrombopoietin receptor agonists. In cases in which the platelet count is below this threshold, the decision to initiate anticoagulation should be individualized, taking into account the severity of thrombocytopenia, the risk of thrombosis, and the bleeding risk. Both low molecular weight heparin (LMWH) and vitamin K antagonists have been used, while data on DOACs remain limited in this population [75].

In patients with von Willebrand disease (VWD) who develop VTE, management is also challenging due to the increased bleeding risk inherent to their underlying condition. Anticoagulant therapy can be administered to VWD patients, but it should always be accompanied by replacement therapy to maintain adequate levels of von Willebrand factor (VWF) and factor VIII (FVIII). Specifically, during anticoagulation, VWF levels should be kept above 30 IU/dl and above 50 IU/dl in the case of major bleeding or invasive procedures. The choice of anticoagulant, whether LMWH, DOACs, or vitamin K antagonists, should be individualized based on the patient’s bleeding phenotype, the severity of VWD, and the feasibility of close monitoring and prompt correction of bleeding complications [76].

Close clinical and laboratory monitoring is crucial, with immediate access to replacement therapy in case of bleeding events. Additionally, the importance of a multidisciplinary approach, involving both hematologists and the physicians managing the thrombotic event, is crucial to optimize safety and efficacy. The overarching principle is that anticoagulant therapy should not be withheld when indicated but must always be safeguarded by appropriate replacement therapy and vigilant follow-up [69].

Similarly, anticoagulated IPD patients should be carefully monitored with frequent clinical and laboratory controls. Moreover, in cases of surgery, patients should be closely checked, with frequent drainage device inspections and imaging in case of suspected hemorrhage. IVC filters remain an option for patients for whom the bleeding risk is considered prohibitive and, simultaneously, who are exposed to a high thrombotic risk procedure.

## 9. How We Approach Thromboembolism in IPD Patients

### Thromboprophylaxis to Prevent VTE

**Figure 2:**
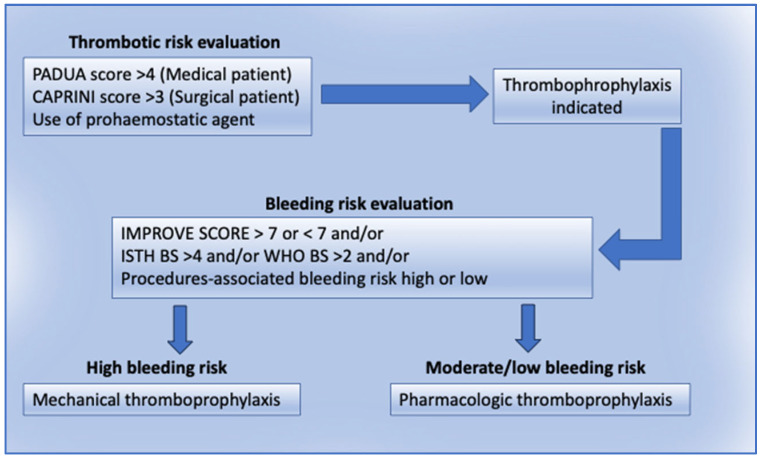
Approach to VTE risk management in IPD.

Currently, no sufficient data are available to support pharmacologic against mechanical prophylaxis in IPD, even if the few available data suggest that both mechanical and pharmacologic thromboprophylaxis may be safe and effective in these patients. The choice should be guided by a careful evaluation of the individual bleeding risk assessed, with the available tools, including the ISTH BAT and the VTE BLEED score, together with the thromboembolic risk, which may be assessed with the scores used in the general population for medical and surgical settings. In case of surgery, the intrinsic bleeding risk associated with the procedure should also be considered, as well as the efficiency of the hemostasis obtained during the intervention. In patients at higher bleeding risk, mechanical thromboprophylaxis should be considered. In patients where the VTE risk is very high (estimated risk > 2%), the use of LMWH should be considered, informing the patient about the pros and cons (Figure 2).

## 10. Future Perspectives and Unmet Needs

Despite advances in the understanding of IPDs and VTE, significant knowledge gaps remain, particularly regarding the optimal prevention and management strategies. In the context of IPD, thromboembolic risk remains a clinical challenge that is still poorly explored and often underestimated. While there is increasing attention to bleeding complications, evidence regarding predictors of thromboembolic events in patients with inherited bleeding disorders, and in particular in IPD, is limited and fragmented [77,78,79]. This situation generates numerous clinical uncertainties in daily practice, making it difficult for clinicians to accurately identify at-risk patients and implement targeted preventive strategies. It is therefore crucial to promote studies to clarify that clinical, laboratory, or genetic elements can guide a more precise assessment of the thrombotic risk in IPD, with the aim of optimizing management and improving patient outcomes. Prospective studies, international registries, and collaborative research efforts are strongly needed to develop evidence-based guidelines tailored to the unique needs of this population.

## 11. Conclusions

IPD patients face a complex interplay of bleeding and thrombotic risks, particularly in high-risk situations such as surgery. An individualized, multidisciplinary approach to their management is essential, and further clinical research is urgently needed to inform clinical practice and improve patient outcomes.

## Figures and Tables

**Table 1:** Inherited platelet disorders (IPD) with reported venous thromboembolic events.

Disorder	Genetic Defect/Main Feature	Bleeding Risk	VTE Cases Reported
Glanzmann Thrombasthenia (GT)	αIIbβ3 integrin deficiency	High	10 cases [27,30]
Bernard-Soulier Syndrome	GPIb-IX-V complex deficiency	High	1 case [30]
MYH9-Related Disorders	MYH9 gene variation	Moderate	2 cases [27]

**Table 2:** Classification of inherited platelet disorder (IPD) according to bleeding phenotype [56].

Mild (ISTH BAT BS 0–4)	Moderate (ISTH BAT BS 5–10)	Severe (ISTH BAT BS >11)
Defect of the TP receptor Defects of collagen receptors MYH9-related disease ANKRD26-related thrombocytopenia Monoallelic Bernard-Soulier syndrome ETV6 -related disease ACTN1-related thrombocytopenia X-linked thrombocytopenia CYCS-related thrombocytopenia TUBB1-related thrombocytopenia	δ -storage pool deficiency Bernard Soulier syndrome_Biallelic Primary secretion defect Familial platelet disorder and predisposition to acute myelogenous leukemia Hermansky-Pudlak syndrome Defect of the P2Y12 Purinergic Receptor Combined alpha-delta granule deficiency ITGA2B/ITGB3-related thrombocytopenia Thrombocytopenia with absent radii	GT Gray platelet syndrome Quebec platelet disorder CalDAG-GEFI defect Paris-Trousseau syndrome cPLA2 deficiency Platelet-type Von Willebrand Disease, GATA1-related diseases Wiskott-Aldrich syndrome

## Data Availability

No new data were created or analyzed in this study. Data sharing is not applicable to this article.

## Abbreviations

The following abbreviations are used in this manuscript:

ACCP	American College of Chest Physician
LMWH	Low molecular weight heparin
WHO	World health organization
IPD	Inherited platelet disorders
IVC	Inferior vena cava
VTE	Venous thromboembolism
ISTH	International society of thrombosis and haemostasis
DOAC	Direct oral anticoagulant
